# Reduced-dose WBRT combined with SRS for 1–4 brain metastases aiming at minimizing neurocognitive function deterioration without compromising brain tumor control

**DOI:** 10.1016/j.ctro.2022.09.005

**Published:** 2022-09-27

**Authors:** Toshimichi Nakano, Hidefumi Aoyama, Shunsuke Onodera, Hiroshi Igaki, Yasuo Matsumoto, Ayae Kanemoto, Shigetoshi Shimamoto, Masayuki Matsuo, Hidekazu Tanaka, Natsuo Oya, Tomohiko Matsuyama, Atsushi Ohta, Katsuya Maruyama, Takahiro Tanaka, Nobutaka Kitamura, Kohei Akazawa, Katsuya Maebayashi, for the Japanese Radiation Oncology Study Group 13-1 (JROSG13-1) Investigators

**Affiliations:** a Department of Radiology and Radiation Oncology, Niigata University Graduate School of Medical and Dental Sciences, Niigata, Japan; b Department of Radiation Oncology, Hokkaido University Faculty of Medicine, Sapporo, Japan; c Department of Radiation Oncology, Hokkaido Cancer Center, Sapporo, Japan; d Department of Radiation Oncology, National Cancer Center Hospital, Tokyo, Japan; e Department of Radiation Oncology, Niigata Cancer Center Hospital, Niigata, Japan; f Department of Radiation Oncology, Osaka General Medical Center, Osaka, Japan; g Department of Radiology, Gifu University Graduate School of Medicine, Gifu, Japan; h Department of Radiation Oncology, Yamaguchi University Graduate School of Medicine, Ube, Japan; i Department of Radiation Oncology, Faculty of Life Sciences, Kumamoto University, Kumamoto, Japan; j Clinical and Translational Research Center, Niigata University Medical and Dental Hospital, Niigata, Japan; k Department of Medical Informatics, Niigata University Medical and Dental Hospital, Niigata, Japan; l Division of Radiation Oncology, Nippon Medical School Hospital, Tokyo, Japan

**Keywords:** Brain metastases, Cognition, Recurrence, Radiation, Whole brain, Stereotactic radiosurgery

## Abstract

**Background and purpose:**

To minimize cognitive decline without increasing brain tumor recurrence (BTR) by reduced-dose whole-brain radiotherapy (RD-WBRT) (25 Gy, 10 fractions) + stereotactic radiosurgery (SRS) in patients with ≤ 4 brain metastases.

**Materials and methods:**

Eligible patients with ≤ 4 brain metastases on contrast-enhanced MRI and Karnofsky Performance Status ≥ 70. The primary endpoint was the non-inferiority of BTR at distant sites in the brain (BTR-distant)-free survival at 6 months compared to that of the standard dose (SD)-WBRT (30 Gy, 10 fractions) + SRS arm in a randomized clinical trial (JROSG99-1) of SRS with/without SD-WBRT. Secondary endpoints included BTR at any brain sites (BTR-all) and neurocognitive function assessed by a six-test standardized battery.

**Results:**

Forty patients from seven institutions were enrolled (median age 69 years). The primary tumor site was a lung in 28 patients; 20 patients had a solitary brain metastasis. The median survival time was 19.0 months (95 %CI: 13.8 %–27.5 %). The BTR-distant-free survival at 6 months was 76.9 % (59.5 %–87.7 %), which is comparable to that of historical control although predetermined non-inferiority (>71 %) could not be confirmed (p = 0.16). The cumulative incidence of BTR-all at 6 months accounting for the competing risk of death was 23.0 % (11.4–37.1), which was not worse than that of historical control (p = 0.774). The frequency of the cumulative incidence of persistent cognitive decline at 6 months was 48.6 % under the [>2.0 SD in ≥ 1 test] definition.

**Conclusions:**

RD-WBRT may yield comparable intracranial tumor control when combined with SRS, and may reduce the risk of neurocognitive decline compared to that after SD-WBRT.

## Highlights

• The addition of whole-brain radiotherapy (WBRT) to stereotactic radiosurgery (SRS) reduces the risk of brain tumor recurrence but standard-dose WBRT (SD-WBRT) accompanies the risk of neurocognitive decline.
• Reduced-dose WBRT (RD-WBRT) combined with SRS provides intracranial tumor control rate comparable to that after SD-WBRT + SRS.
• RD-WBRT could reduce the risk of neurocognitive decline compared to that after SD-WBRT.

## Introduction

### Background

Brain metastasis is the most common brain tumor, as it develops in 20 % – 40 % of patients with systemic cancers. The number of patients diagnosed with brain metastases has been constantly increasing, due in part to the improvement of systemic therapy and the increasing quality and prevalence of MRI in recent years.[1] The standard treatment for brain metastases has been whole-brain radiation therapy (WBRT), but there has also been concern about the cognitive deterioration of patients as a result of the toxicity of WBRT. Treatment with stereotactic radiosurgery (SRS) without WBRT is thus becoming more widely used for patients whose number of brain metastases is limited to 3–4.[2], [3] However, the avoidance of WBRT results in a higher risk of brain tumor recurrence (BTR) at distant sites in the brain (BTR-distant) with or without BTR at local sites that received SRS (BTR-all). More importantly, the higher risk of BTR could translate to an impairment of overall survival in some subsets of patients.[4] In addition, there remains large regions of the world where frequent monitoring by enhanced-MRI after SRS-alone or advanced forms of radiation therapy such as intensity modulated radiation therapy including hippocampal-avoidance [HA]-WBRT are not commonly available. The optimal use of WBRT thus remains to be determined.

The risk of developing one or more radiation-induced late adverse effects, including cognitive decline, is closely related to the total radiation dose and the dose-per-fraction. The current standard dose-fractionation regimens of WBRT, such as 30 Gy in 10 fractions, were established in the 1970s – 80s when MRI and stereotactic radiosurgery (SRS) were not commonly available, and the primary treatment goal of WBRT was its therapeutic effect on already visualized metastases rather than non-visualized micro-metastases. Today, both WBRT and SRS are readily available worldwide, and thus we considered that the role of WBRT could be limited to merely avoiding the progression of non-visualized micro-metastases or slightly enhancing the treatment effect for already visualized metastases (especially large metastases).

Reduced dose (RD)-WBRT, such as 25 Gy in 10 fractions, was used in the clinical trials examining the role of prophylactic cranial irradiation (PCI) for limited-stage small cell lung cancer (SCLC)[5] or locally advanced non-small-cell lung cancer,[6] and the incidence of cognitive decline in these scenarios is reported to be significantly lower than that by standard-dose WBRT (SD-WBRT).[5] In addition, in a single-arm study conducted using 25 Gy/10 fractions in patients with SCLC, transient decreases were observed in executive function and language after PCI, but the decreases improved to the pretreatment levels in the long term.[7]

We conducted the present non-randomized, single-arm study to determine whether the combination of RD-WBRT and SRS could be used to minimize the risk of cognitive decline without compromising the brain tumor control for patients with 1–4 brain metastases.

## Patients and methods

### Study design and patients

This was a multi-institutional phase II study by the Japanese Radiation Oncology Study Group (JROSG 13–1). Adult patients (20–80 years old) with 1–4 brain metastases, all of which were ≤ 3 cm in diameter, were eligible for the trial. The eligibility criteria also included a Karnofsky Performance Status (KPS) ≥ 70 and pathological confirmation of an extracranial tumor site. The exclusion criteria included a past history of surgery or radiation to the brain, the presence of metastasis to the brainstem or leptomeningeal dissemination, and inability to take cognitive function tests or quality of life (QOL) surveys. Brain metastases from small cell cancers, germ cell tumors, or lymphoma were also excluded. Each participating institution provided institutional review board approval, and each patient provided written informed consent. This trial is registered with the UMIN Clinical Trial Registry (UMIN000009055).

### Procedure

The radiation dose of single-fraction SRS (SF-SRS) prescribed to the 95 % of the gross tumor volume defined as the enhanced area on MRI was 22–24 Gy for lesions ≤ 2 cm and 18–22 Gy for lesions >2 cm. The use of hypo-fractionated SRS (HF-SRS), which has an effect that is biologically identical to that of various protocols of SF-SRS, such as 28–35 Gy in 4 fractions or 26–30 Gy in 3 fractions, was allowed. RD-WBRT (25 Gy in 10 fractions) was started within 1 week after the final date of the patient's SRS.

### End points

This study's primary endpoint was the BTR-distant-free survival of patients at 6 months after the completion of radiation therapy. The secondary endpoints included overall survival (OS), local tumor control, cognitive functional change, radiation-related adverse effects, and the cause of death. The cumulative incidences of BTR-distant and BTR-all were estimated by the competing risk method to account for the competing risk of death. Gray's test was used to test for significant differences in the cumulative incidence of BTR-distant and BTR-all.

Contrast-enhanced MRI was taken at baseline and 4, 6, 9, and 12 months and every 6 months thereafter. The standardized neuropsychological test battery[8] was used, which included the Hopkins Verbal Learning Test Revised (HVLT-R) for memory (both immediate and delayed recall and recognition), the Controlled Oral Word Association Test (COWA) for language/verbal fluency, the Trail Making Test Part A (TMT-A) for visual and spatial scanning, attention, sequencing, and speed, and the Trail Making Test Part B (TMT-B) for executive/frontal lobe skills at baseline and 4, 8, and 12 months and every 6 months thereafter. The quality-of-life measures included the European Organization for Research and Treatment of Cancer (EORTC) Quality-of-Life Questionnaire (QLQ-C30) and the Brain Cancer Module 20 (BN20).[5] All treatment-related toxicities and adverse events were recorded according to the National Cancer Institute Common Terminology Criteria for Adverse Events (CTCAE) version 4.0.

### Sample size and statistical analyses

The target accrual was 40 patients with 33 as the number of occurred events. This target was calculated using the 95 % confidence interval (95 %CI) for an exponential model, based on having 80 % power to detect the non-inferiority of RD-WBRT combined with stereotactic radiosurgery (SRS) of JROSG13-1 compared to that of SD-WBRT (30 Gy in 10 fractions) in the JROSG99-1 study[2] (a randomized clinical trial [RCT] between SRS + SD-WBRT and SRS alone) with a one-sided significance level of 0.05 assuming that the 6-month BTR-distant-free survival of the JROSG99-1 study was 81 %, and defining non-inferiority as the same 6-month disease-free survival of >71 %.[9] We performed a one-sided test for the non-inferiority of the JROSG13-1 study by comparing the observed 6-month BTR-distant-free survival rate with a margin of 10 % (i.e., a null hypothesis that the hazard ratio of the JROSG13-1 study was ≥ 1.625). In addition, a comparison of BTR-distant-free survivals accounting for the entire follow-up period of patients in each study was also conducted as a reference.

The statistical analyses were performed with SAS software, ver. 9.4 (SAS Institute, Cary, NC) and EZR (a modified version of R commander).[10] The cumulative incidences of BTR-distant and BTR-all were estimated by the competing risk method to account for the competing risk of death, and the statistical difference derived from the results of the JROSG99-1 study was compared by Gray's test. The OS and BTR results of the JROSG99-1 trial[2] were calculated from the date of the last day of treatment for the comparison with the results of the present study.

## Results

### Study patients

Between April 2012 and November 2018, 40 patients were enrolled at seven participating institutions in Japan. The data were fixed in November 2019. All analyses were undertaken after all patients had been potentially followed for ≥ 12 months. All patients completed the pretreatment cognitive and patient-reported QOL assessment. The patients' baseline characteristics are summarized in Table 1: there were 22 males and 18 females, and the median age was 69 years (range 43–80 yrs). The primary tumor site was a lung in 28 patients (70 %). All 9 patients with EGFR- or ALK-positive lung adenocarcinomas received a treatment with tyrosine kinase inhibitors (TKIs) before and/or after brain radiation therapy.

**Table 1:** Characteristics of the 40 patients at the patient and lesion levels

Characteristics		
Patient level (N = 40)		
Age, years	Mean (SD)	68.3 (8.1)
	Median	69
	Range:	43–80
	18-59	7 (17.5 %)
	60-69	15 (37.5 %)
	70-80	18 (45 %)

Gender	Male	22
	Female	18

Primary tumor	Lung, adenocarcinoma	24
	(EGFR or ALK positive)*	(9)
	(EGFR or ALK negative)	(15)
	Lung, non-adenocarcinoma	4
	(Squamous-cell carcinoma)	(2)
	(Others)	(2)
	Breast	2
	Kidney	2
	Colon	4
	Bladder	2
	Ovary	2

KPS	100	15
	90	16
	80	5
	70	4

Status of Primary cancer	Controlled	23
	Not-controlled	17

Extracranial metastases	Absent	18
	Present	22

Number of brain metastases	1	21
	2	10
	3	5
	4	4

DS-GPA	0-1.0	2
	1.5-2.0	11
	2.5-3.0	19
	3.5-4.0	8

Neurologic symptom	Symptomatic	16
	Asymptomatic	24

Cognitive test score**		
Z-score mean (SD)	HVLT-R total recall	-1.27 (1.24)
	HVLT-R delay recall	-1.49 (1.25)
	HVLT-R delay recognition	-0.76(1.23)
	TMT-A	-0.78 (1.42)
	TMT-B	-1.31 (1.92)
	COWA	-0.27 (1.14)

Lesion level (N = 73)		
Size, maximum diameter (mm)	Median	11 mm
	<10 mm	27
	10-19 mm	25
	20-30 mm	21

Radiation method	Single-fraction SRS	36
	Hypo-fractionated SRS	37

Abbreviations: EGFR, Epidermal Growth Factor Receptor; ALK, Anaplastic Lymphoma Kinase; TKI, Tyrosine Kinase Inhibitor; KPS, Karnofsky Performance Status; DS-GPA, Disease-specific Graded Prognostic Assessment; HVLT-R, Hopkins Verbal Learning Test Revised; TMT, Trail Making Test; COWA, Controlled Oral Word Association.

* All patients with EGFR or ALK positive lung-adenocarcinoma received Tyrosine Kinase Inhibitor (TKI) treatment.

** Cognitive tests are reported as standardized score (z-score, transformed so that higher scores indicate better cognitive performance) : (patient value – published-norm mean value)/published-norm standard deviation value.

SF-SRS, 4-fraction HF-SRS, and 3-fraction HF-SRS were used in 36, 29, and 8 lesions, respectively. HF-SRS rather than SF-SRS was significantly more often applied to large metastases (≥1.5 cm) than smaller ones: 85.7 % (24/28) vs. 28.8 % (13/45) (p = 0.000002). The median prescribed radiation doses of the SF-SRS, 4-fraction HF-SRS, and 3-fraction HF-SRS were 22 Gy (range 18–23.7), 32.0 Gy (20–34.2), and 29.4 Gy (25.1–29.8) respectively. The median follow-up time was 16.3 months (range 2.5–49.7).

### Overall survival and brain tumor recurrence

**Fig. A1:**
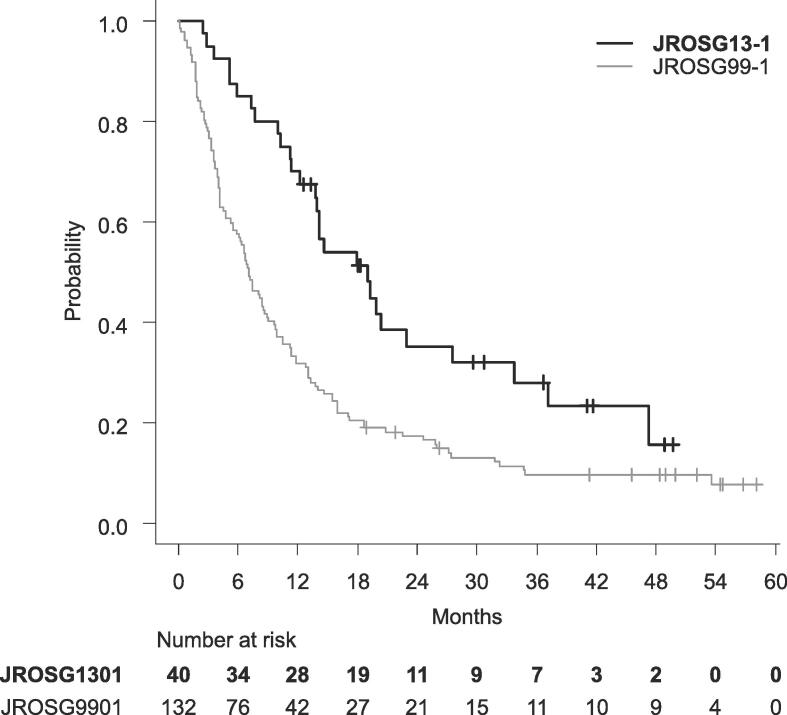
Overall survivals of the present study (JROSG13-1) and the historical data (JROSG99-1)

The median survival time (MST) was 19.0 months (95 %CI: 13.8–27.5), which was significantly longer than that of the JROSG99-1 (median 7.1, 95 %CI: 5.5–8.7) (log-rank, p = 0.0004) (Fig. A1), even though significantly older patients were included in the present study than in the JROSG99-1 (Table A1). In univariate analyses, KPS (90-100 vs. 70-80, p = 0.034), age (<70 vs. ≥ 70, p = 0.019), and the diagnosis-specific graded prognostic assessment (DS-GPA) (3.5-4.0 vs. 2.5-3.0 vs. 0-2.0, p = 0.017) were associated with better OS. Patients with EGFR- or ALK-positive lung-adenocarcinoma showed a trend of better OS compared to those with other histopathologies (MST: 37.2 months vs. 14.6 months, p = 0.077) (Table A2).

**Fig. A2:**
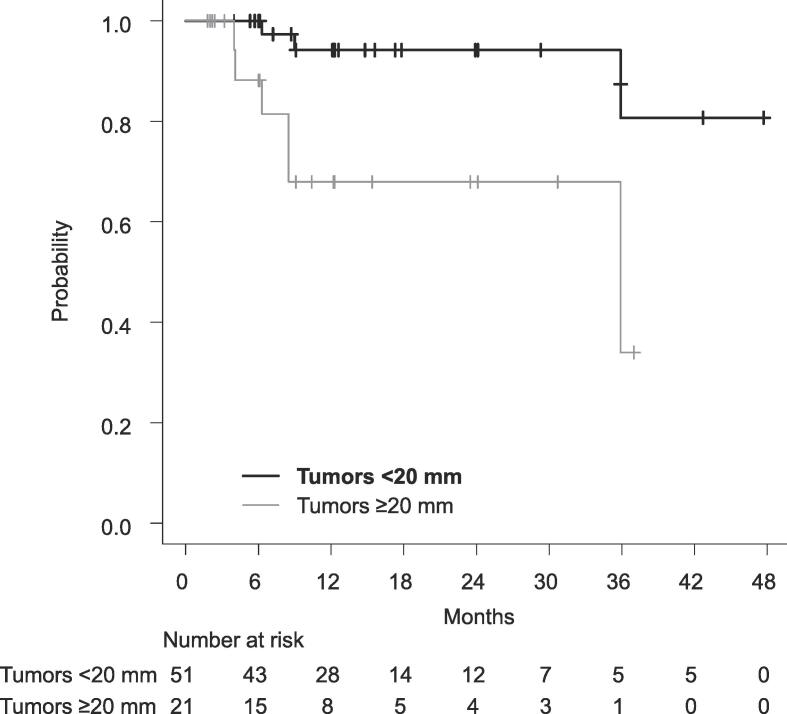
Local tumor control according to maximum tumor diameter

Follow-up enhanced MRI was available in all but one of the 40 patients, and 72 of the 73 treated lesions were evaluable with enhanced MRI at least once. Local tumor progression was observed in 9 of 72 lesions among 7 patients. The 12-month local tumor control rate was 86.7 % (95 %CI: 73.9–93.5) overall. Larger tumor diameter (≥20 mm) was associated with a lower control rate compared to that of smaller tumor diameter (≤19 mm): 67.9 % (95 %CI: 38.9–85.3) versus 94.2 % (95 %CI: 78.5–98.5) at 12 months, respectively (log-rank, p = 0.003) (Table 2, Fig. A2).

**Table 2:** Local tumor control of JROSG13-1 and JROSG99-1 according to the maximum tumor diameter

				Time point % (95 % CI)			
	Maximum diameter	Treatment	N	6 months	12 months	24 months	*p*
JROSG13-1	All	RD-WBRT + SRS	72	96.9 (88.1–99.2)	86.7 (73.9–93.5)	86.7 (73.9–93.5)	
(present study)	≥15 mm	RD-WBRT + SRS	28	91.5 (70.0–97.8)	75.3 % (50.1–89.0)	75.3 % (50.1–89.0)	0.009
	<15 mm	RD-WBRT + SRS	44	100 %	93.4 % (75.9–98.3)	93.4 % (75.9–98.3)	
	≥20 mm	RD-WBRT + SRS	21	88.2 (60.6–96.9)	67.9 % (38.9–85.3)	67.9 % (38.9–85.3)	0.003
	<20 mm	RD-WBRT + SRS	51	100 %	94.2 % (78.5–98.5)	94.2 % (78.5–98.5)	

JROSG99-1	All	All	205	87.4 % (80.5–92.0)	80.2 % (71.3–86.6)	71.0 % (58.4–80.4)	
[2]	All	SD-WBRT + SRS	90	91.1 % (79.8–96.2)	88.6 % (76.0–94.8)	88.6 % (76.0–94.8)	0.004
		SRS-alone	115	84.8 % (74.9–91.0)	73.2 % (59.2–83.0)	51.2 % (29.8–69.1)	
	≥15 mm	SD-WBRT + SRS	43	84.9 % (64.0–94.2)	79.2 % (56.0–91.1)	79.2 % (56.0–91.1)	0.042
		SRS-alone	50	83.0 % (62.2–92.9)	60.7 % (32.3–8.02)	16.2 % (9.5–49.1)	
	<15 mm	SD-WBRT + SRS	47	96.4 % (77.2–99.5)	96.4 % (77.2–99.5)	96.4 % (77.2–99.5)	0.023
		SRS-alone	65	88.2 % (75.5–94.6)	81.1 % (64.3–90.6)	74.9 % (53.7–87.4)	
	≥20 mm	SD-WBRT + SRS	30	90.5 % (66.7–97.6)	81.5 % (50.1–94.1)	81.5 % (50.1–94.1)	0.06
		SRS-alone	30	83.0 % (53.1–94.7)	59.8 % (23.3–83.4)	19.9 % (1.0–56.7)	
	<20 mm	SD-WBRT + SRS	60	91.9 % (76.9–97.3)	91.9 % (76.9–97.3)	91.9 % (76.9–97.3)	0.013
		SRS-alone	85	85.4 % (74.3–92.0)	76.6 % (61.5–86.4)	62.8 % (38.7–79.7)	

Abbreviations: RD-WBRT, reduced-dose whole brain radiation therapy; SD-WBRT, standard-dose whole brain radiation therapy; SRS, stereotactic radiosurgery

**Fig. A3:**
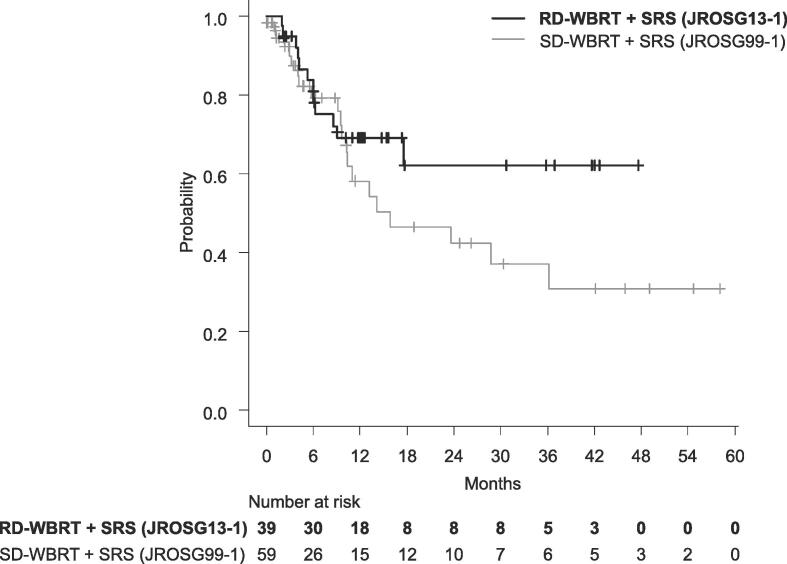
BTR-distant free survival of the present study (JROSG13-1) and the historical data (JROSG99-1)

Regarding the primary endpoint, BTR-distant was observed in 12 patients. The BTR-distant-free survival at 6 months was 76.9 % (95 %CI: 59.5–87.7) (Fig. A3), and therefore predetermined non-inferiority (>71 %) could not be confirmed (p = 0.16). However, non-inferiority was observed in the test accounting for the entire follow-up period (p = 0.004).

**Fig. 1:**
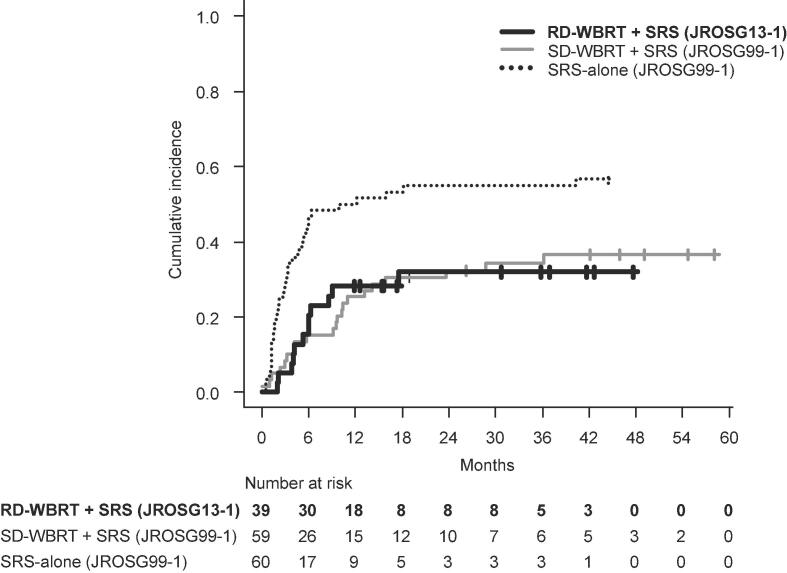
Cumulative incidence of BTR-distant in the present study (JROSG13-1) and the historical data (JROSG99-1)

**Fig. A4:**
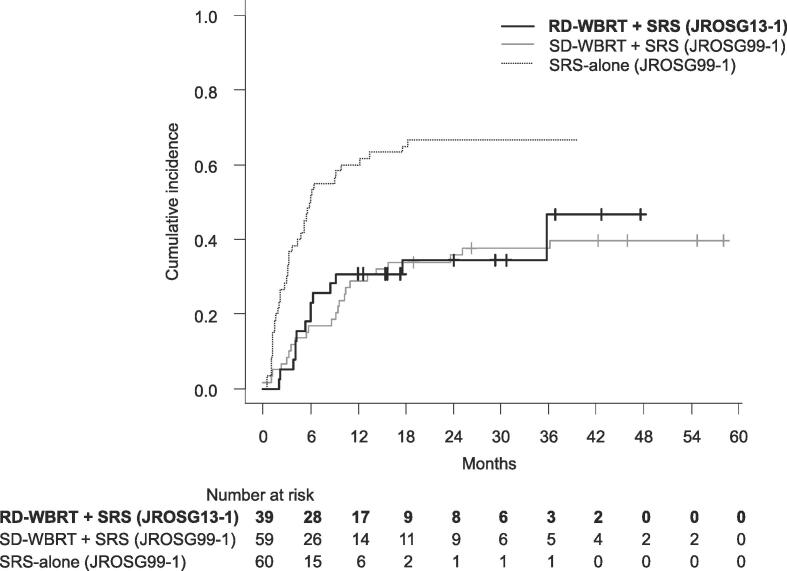
Cumulative incidence of BTR-all in the present study (JROSG13-1) and historical data (JROSG99-1)

The cumulative incidences of BTR-distant at 6 and 12 months were 20.5 % (95 %CI: 9.6–34.2) and 28.2 % (95 %CI: 15.3–42.7), respectively, and these values were comparable to those of the SD-WBRT arm of JROSG 99-1, which were 15.2 % (95 %CI: 7.5–25.5) and 32.3 % (95 %CI: 20.9–44.4), respectively (p = 0.846) (Fig. A4, Table 3). The cumulative incidences of BTR-all at 6 and 12 months were 23.0 % (95 %CI: 11.4–37.1) and 30.7 % (95 %CI: 17.3–45.4), which were comparable to those of the SD-WBRT arm of the JROSG 99-1 study at 16.9 % (95 %CI: 8.7–27.5) and 28.8 % (95 %CI:18.0–40.6), respectively (p = 0.774) (Fig. 1b). In univariate analyses, none of the factors, including the histopathological factors (EGFR/ALK-positive lung-adenocarcinoma vs. others), exhibited statistical significance, with the exception of extracranial metastatic status. Patients with the existence of extracranial metastases showed a trend of higher cumulative incidence for BTR-distant (p = 0.080) and BTR-all (p = 0.040) compared with those without extracranial metastasis

**Table 3:** Cumulative incidence of brain tumor recurrence (BTR) in the JROSG13-1, JROSG99-1 and N0574 trials

Cumulative incidence	Treatment	6 months	12 months
BTR-distant	RD-WBRT + SRS (present study)	20.5 % (9.6-34.2)	28.2 % (15.3-42.7)
	SD-WBRT + SRS (JROSG99-1) [2]	15.2 % (7.5-25.5)	25.4 % (15.2-37.0)
	SD-WBRT + SRS (N0574) [3]	5.3 % (0.7-9.7)	7.5 % (2.0-12.7)
	SRS-alone (JROSG99-1) [2]	46.6 % (33.7-58.6)	50.0 % (36.8-61.8)
	SRS-alone (N-0574) [3]	22.9 % (14.4-30.5)	30.0 % (20.5-38.3)

BTR-all	RD-WBRT + SRS (present study)	23.0 % (11.4-37.1)	30.7 (17.3-45.4)
	SD-WBRT + SRS (JROSG99-1) [2]	16.9 % (8.7-27.5)	28.8 % (18.0-40.6)
	SD-WBRT + SRS (N0574) [3]	11.6 % (4.9-17.8)	15.0 % (7.4-21.9)
	SRS-alone (JROSG99-1) [2]	51.6 % (38.4-63.4)	60.0 % (46.5-71.1)
	SRS-alone (N-0574) [3]	35.3 % (25.5-43.9)	49.5 % (38.9-58.3)

Abbreviations: RD-WBRT, reduced-dose whole-brain radiation therapy; SD-WBRT, standard-dose whole-brain radiation therapy; SRS, stereotactic radiosurgery

## Neurocognitive function, quality of life, and adverse events

The patients' compliance values for the cognitive tests were 100 % at baseline, 97.3 % (36/37 survivors) at 4 months, 81.3 % (26/32) at 8 months, and 70.4 % (19/27) at 12 months. The cumulative incidence of persistent neurocognitive decline is summarized in Table 4. The values of decline at 6 months varied from 48.6 % to 75.0 % depending on the definition of decline adopted. The mean QOL values at each time point are summarized in Table A5. Significantimprovementaftertreatmentwasobservedinemotionalfunctioning and social functioning; otherwise, a general trend of maintenance of scores up to 12 months compared to the baseline scores was observed. Regarding toxicities, Grade 3 or greater treatment-related adverse events were observed in 3 patients (Table A6). Grade 2 or greater radiation necrosis was observed in 3 patients.

**Table 4:** Neurocognitive decline rate in the present study and publications

Trial name	Treatment	Age (years)	Definition of decline	Time point (months)	Decline rate
JROSG13-1	RD-WBRT + SRS	Mean 68.3 (SD 8.1)	>1.0 SD in ≥ 1 test	6	75.0 %
(present study)		Median 69 (range 43-80)	>1.5 SD in ≥ 1 test		59.6 %
			>1.5 SD in ≥ 2 test		42.6 %
			>2.0 SD in ≥ 1 test		48.6 %
			>RCI in ≥ 1 test		56.7 %
			>2.0 SD or RCI in ≥ 1 test		59.7 %

NCCTG N0574 [3]	SD-WBRT + SRS	Mean 61.4 (SD 10.6)	>1.0 SD in ≥ 1 test	3	91.7 %
			>1.5 SD in ≥ 2 tests		45.8 %
			>2.0 SD in ≥ 1 test		72.9 %
	SRS-alone	Mean 59.8 (SD 10.4)	>1.0 SD in ≥ 1 test	3	63.5 %
			>1.5 SD in ≥ 2 tests		19.0 %
			>2.0 SD in ≥ 1 test		42.9 %

NCCTG N107C/CEC3 [11]	Surgery + SD-WBRT	Median 62 (IQR 54-68)	>1.0 SD in ≥ 1 test	6	85.0 %
			>1.5 SD in ≥ 2 test		52.1 %
			>2.0 SD in ≥ 1 test		50.0 %
	Surgery + SRS	Median 61 (IQR 54-66)	>1.0 SD in ≥ 1 test	6	52.0 %
			>1.5 SD in ≥ 2 test		14.8 %
			>2.0 SD in ≥ 1 test		27.8 %

RTOG 0614 [13]	SD-WBRT + memantine	Median 60 (range 31-84)	>2.0 SD or RCI in ≥ 1 test	6	53.5 %
	SD-WBRT + placebo	Median 59 (range 29-86)	>2.0 SD or RCI in ≥ 1 test	6	64.9 %

NRG CC001 [14]	SD-WBRT + memantine	Median 61 (range 20-88)	>RCI in ≥ 1 test	6	68.2 %
	SD-HA-WBRT + memantine	Median 62 (range 27-91)	>RCI in ≥ 1 test	6	59.5 %

SAKK 15/12 [12]	RD-HA-WBRT	Median 62.5 (range 34-75)	>1.0 SE in ≥ 1 test	6	65.8 %

## Discussion

The cumulative incidences of BTR-distant and BTR-all after RD-WBRT in the present study were comparable to the corresponding values after SD-WBRT in the JROSG 99-1 trial and were significantly lower than those after SRS-alone. Therefore, the dose-fractionation of WBRT when combined with SRS could be safely reduced to 25 Gy in 10 fractions without increasing the risks of BTR-distant and BTR-all.

The incidences of cognitive decline in this study according to the different definitions of cognitive decline are summarized in Table 4, and we compared these values to the results of previous brain metastasis trials in which cognitive function after radiation was assessed by the same standardized cognitive battery.[8] The definitions of cognitive decline are not standardized and they differ among clinical trials; results should be carefully interpreted when they are compared with those of other trials, taking into account the definition of cognitive decline used.[11]

**Fig. 2:**
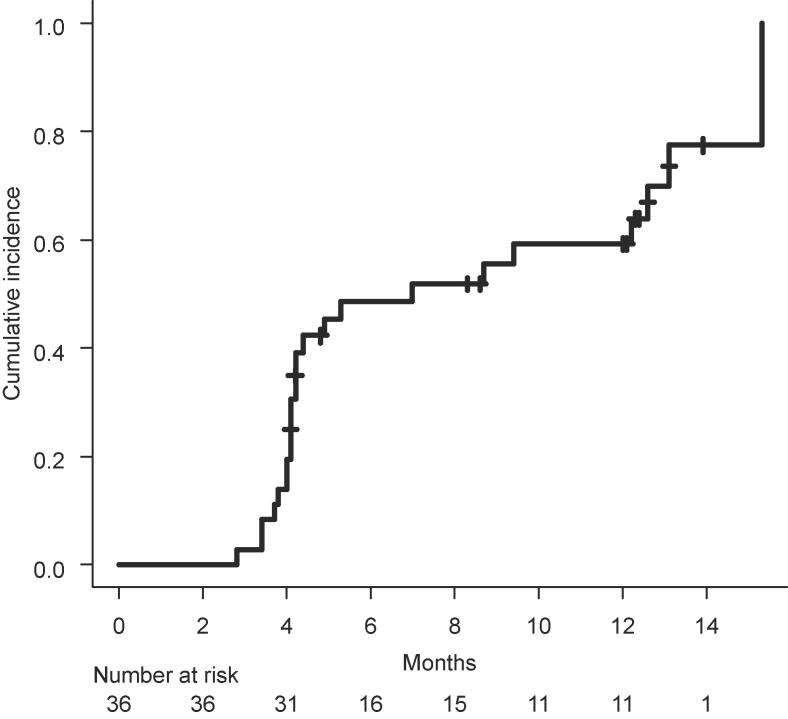
Cumulative incidence of persistent neurocognitive decline defined as “>2.0 SD in ≥ 1 test”

When we defined decline as [>2.0 SD in ≥ 1 test] in the present study, the rate of decline was 48.6 % (Fig. 2). In the N0574 trial comparing SD-WBRT + SRS and SRS-alone,[3] the rates of decline were 72.9 % and 42.9 %, respectively. The decline rate in the present study was thus much lower than that in the SD-WBRT + SRS group in the N0574 trial, and it was close to that in the SRS-alone group.

When we used [>1.0 SD in ≥ 1 test] as the definition of decline, the cognitive decline rate was 75.0 %. Similar definitions were used in the N107C/CEC3 trial (Surgery + SD-WBRT vs. Surgery + SRS),[12] the N0574 trial,[3] and the SAKK 15/12 trial (Hippocampal-avoidance [HA]-RD-WBRT).[13] The rates of cognitive decline in these studies were as follows: 85 % (N107C/CEC3) and 91.7 % (N0574) in the SD-WBRT group, 52 % (N107C/CEC3) and 63.5 % (N0574) in the SRS-alone group, and 65.8 % (SAKK15/12) in the HA-RD-WBRT group. Compared to those numbers, the decline rate in our present investigation is somewhere between SD-WBRT and the others. In addition, it is noteworthy that Vees et al. concluded in the SAKK15/12 trial that the rate of neurocognitive decline after HA-RD-WBRT was not significantly different from that of RD-WBRT.[13] However, it should be noted that the [>1.0 SD in ≥ 1 test] criterion has high sensitivity but low specificity.[11]

**Fig. A5:**
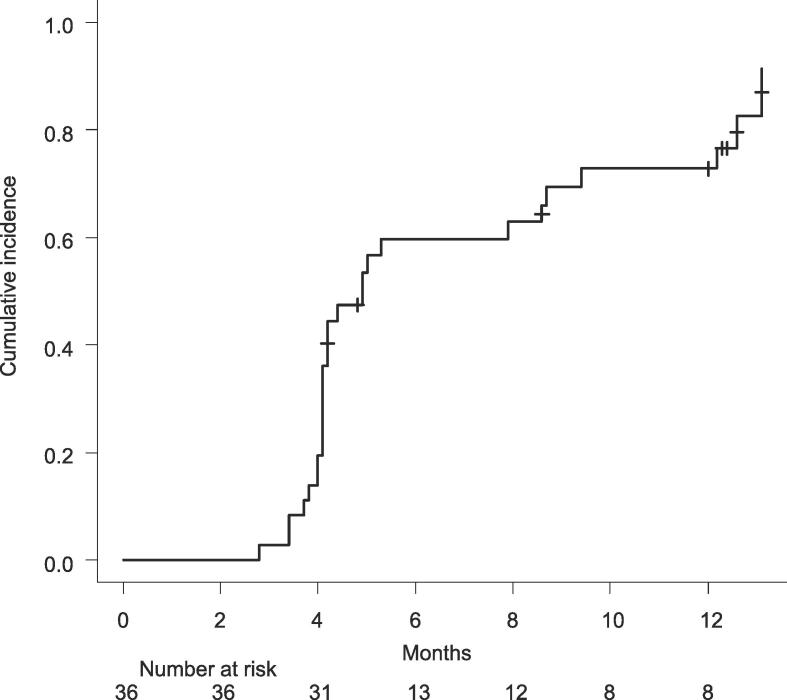
Cumulative incidence of persistent neurocognitive decline defined as “>2.0 SD or RCI in ≥ 1 test”

When we used [>2.0 SD or > RCI (reliable change index) in ≥ 1 test] as the definition of cognitive decline, the decline rate was 59.7 % (Fig. A5). This definition was also used in the RTOG0614 trial (SD-WBRT + memantine vs. SD-WBRT + placebo).[14] The rate of cognitive decline in the present study was similar to the 64.9 % after SD-WBRT with placebo and 53.5 % after SD-WBRT with memantine in the RTOG0614 trial. In the NRG CC001 trial, the definition [>RCI in ≥ 1 test] was used as the definition of cognitive decline, and the decline rate was 68.5 % after SD-WBRT + memantine and 59.5 % after HA-SD-WBRT + memantine.[15] The decline rate of 56.7 % in the present study seems similar to that after HA-SD-WBRT + memantine in the NRG CC001 trial. However, the decline rate in the present study under [>1.5SD in ≥ 2 test] was 42.6 %, which is higher than that after SRS-alone, i.e., 19.0 % (N0574) and 14.8 % (NCCTG N107C/CTC3). It would thus be reasonable to speculate that RD-WBRT could reduce the risk of cognitive decline compared to SD-WBRT. However, the risk of cognitive decline due to a toxic effect of WBRT remains.

In addition to the dose-fractionation schedule of WBRT, age is another factor that could strongly affect the rate of decline in patients' cognitive function.[5] The median age of the present patients was rather high at 69 years, and it was 6–9 years higher than the ages of the patients in the other studies cited herein (Table 4). It is quite likely that the ages of our patient population negatively influenced the cognitive preservation rate. In other words, it may be that the rate of cognitive decline after RD-WBRT would be lower than that after SD-WBRT, even though more elderly patients were registered in this study; this indicates the possibility of RD-WBRT as a new standard WBRT schedule for patients who are indicated for SRS from the viewpoint of the preservation of cognitive function.

As SRS becomes more and more widely adopted, and as new systemic therapies with some efficacy against brain metastases emerge, what are the indications for adding WBRT to SRS? As a patient's prognosis improves, the significance of controlling brain tumors becomes more important in regard to OS as well as the maintenance of QOL.[4] Apart from the reduction of BTR-distant, another important role of WBRT combined with SRS is to enhance the local tumor control compared to that provided by SRS alone. The results of all four of the randomized trials comparing SRS alone and SRS + WBRT demonstrated that not only the BTR-distant-free survival but also the local tumor control rate by SRS + WBRT was significantly higher than that by SRS alone.[2], [3], [16], [17] The significant benefit on local tumor control is especially prominent in medium-to-large tumors (≥15–20 mm).[18], [19] In the above-mentioned JROSG99-1 trial, the local tumor control rates of lesions ≥ 20 mm at 12 months were 81.5 % after SD-WBRT + SRS and 59.8 % after SRS alone, and the discrepancy in the rates expanded further at 24 months to 81.5 % versus 19.9 % (Table 2). The use of hypofractionation might improve local tumor control to some extent compared to single-fraction SRS,[18], [19] but we observed that the combination of HF-SRS and RD-WBRT in the present study provided tumor control comparable to that of SD-WBRT + SRS for tumors < 20 mm and ≥ 20 mm at 12 months (94.2 % vs. 67.9 %), and more importantly, this effect was maintained for the next 12 months. The long-term tumor control observed in this study is especially relevant for patients who can expect a good prognosis (i.e., a median OS of 17 months as achieved in this study), in order to achieve long-term maintenance of QOL while avoiding neurologic death, despite the modest risk of neurocognitive decline. Therefore, RD-WBRT combined with HF-SRS might be particularly indicated for patients with favorable prognosis and harboring medium-to-large brain metastases, since medium-to-large brain metastases would be difficult to control by SRS with or without systemic therapies, or could be refractory to systemic therapies. RD-WBRT plus HF-SRS might also be appropriate for patients in whom frequent monitoring by enhanced-MRI after treatment with SRS alone would be difficult for financial or geometrical reasons.

### Study limitations

Limitations of this study include the single-arm design and the small number of patients (n = 40); therefore, these data cannot be used to conclude the superiority of RD-WBRT + SRS over SRS with or without SD-WBRT in terms of avoiding BTR and the preservation of neurocognitive function. In addition, the effect of the use of TKIs was not negligible, though no significant difference was observed between patients who received TKI-therapy for EGFR/ALK-positive lung-adenocarcinoma and the other patients in terms of either BTR-distant or BTR-all in the present study. Nonetheless, our findings are encouraging and merit further investigation in one of the arms of a prospective randomized study designed to identify the optimal treatment for patients with a limited number of brain metastases.

## Conclusions

By achieving durable brain tumor control, the combination of SRS and RD-WBRT may be an optimal treatment method for patients who are expected to have a good life expectancy and to maintain their QOL. The information obtained in this study will be important for physicians in regions of the world where the routine use of HA-WBRT and/or the administration of memantine for patients with brain metastases is not possible.

## Declaration of Competing Interest

The authors declare that they have no known competing financial interests or personal relationships that could have appeared to influence the work reported in this paper.

## Figures and Tables

**Table A1:** Characteristics of the patients in the present study (JROSG13-1) and the historical data (JROSG99-1)

		JROSG13-1 (N = 40)	JROSG99-1 (N = 132)	P
Characteristics				
Age, years	mean (SD)	68.3 (8.1)	62.3 (11.1)	0.0013
Gender	male/Female	22/18	99/33	0.0153
Primary tumor site	lung/Others	28/12	88/44	0.6935
KPS	90-100/70-80	31/9	78/54	0.0343
Status of primary site	controlled/uncontrolled	23/17	69/63	0.5615
Extracranial mets.	absent/present	18/22	54/78	0.6459
Number of brain mets.	1/≥2	21/19	65/66	0.7497

**Table A2:** Overall survival, univariate analysis

Factors		N	MST, months	95 % CI	*p*
KPS	70-80	9	10.0	2.5-NA	0.034
	90-100	31	19.9	14.2-33.7	

Age	≥70	18	14.2	10-19.9	0.019
	<70	22	33.7	11.4-NA	

Extracranial Metastases	Absent	18	22.9	12.2-NA	0.194
	Present	22	14.2	7.7-20.3	

Primary cancer status	Controlled	23	17.9	11.4-47.2	0.725
	Uncontrolled	17	19.9	7.7-27.5	

DS-GPA	0-2.0	13	10.0	5.1-20.3	0.017
	2.5-3.0	19	19.0	11.4-47.2	
	3.5-4.0	8	37.2	14.2-NA	

Histopathology	EGFR or ALK positive lung-adenocarcinoma*	9	37.2	11.2-NA	0.077
	Others	31	14.6	10.3-22.9	

Number of brain mets	1	21	19.0	10.3-33.7	0.414
	2-4	19	19.3	11.2-37.2	

Sum of tumor volume	<5 cc	26	20.3	14.2-47.2	0.060
	≥5 cc	14	13.9	5.1-19.9	

Max diameter of largest met	<10 mm	9	19.7	2.8-NA	0.734
	10-19 mm	11	17.9	10.3-NA	
	≥20 mm	20	17.0	7.7-33.7	

Abbreviations: EGFR, Epidermal Growth Factor Receptor; ALK, Anaplastic Lymphoma Kinase; TKI, Tyrosine Kinase Inhibitor; KPS, Karnofsky Performance Status; DS-GPA, Disease-specific Graded Prognostic Assessment.

* All patients with EGFR or ALK positive lung-adenocarcinoma received Tyrosine Kinase Inhibitor (TKI) treatment.

**Table A3:** Cumulative incidence of BTR-distant, univariate analysis

Factors		N	6 months (95 % CI)	12 months (95 % CI)	*p*
Extracranial Metastases	Absent	18	11.1 (1.9-29.8)	16.6 (4.1-36.5)	0.080
	Present	21	28.5 (11.7-48.2)	38.0 (18.3-57.8)	

Primary cancer status	Controlled	22	18.1 (5.7-36.3)	22.7 (8.3-41.4)	0.280
	Uncontrolled	17	23.5 (7.3-44.9)	35.2 (14.5-57.0)	

Histopathology	EGFR or ALK positive lung-adenocarcinoma*	9	0	11.1 (0.6-38.8)	0.138
	Others	30	26.6 (12.6-43.0)	33.3 (17.5-50.0)	

Sum of tumor volume	<5 cc	25	16.0 (5.0-32.5)	24.0 (9.8-41.7)	0.583
	≥5 cc	14	28.5 (8.8-52.4)	35.7 (13.0-59.4)	

Max diameter of largest met	<19 mm	19	15.7 (3.9-34.9)	26.3 (9.6-46.8)	0.566
	≥20 mm	20	25.0 (9.1-44.9)	30.0 (12.3-50.1)	

No. of brain mets	1	20	20.0 (6.2-39.3)	25.0 (9.1-44.9)	0.871
	2-4	19	21.0 (6.6-41.0)	31.5 (12.9-52.2)	

Abbreviations: EGFR, Epidermal Growth Factor Receptor; ALK, Anaplastic Lymphoma Kinase.

* All patients with EGFR or ALK positive lung-adenocarcinoma received Tyrosine Kinase Inhibitor (TKI) treatment.

**Table A4:** Cumulative incidence of BTR-all, univariate analysis

Factors		N	6 months (95 % CI)	12 months (95 % CI)	*p*
Extracranial Metastases	Absent	18	11.1 (1.9-29.8)	16.6 (4.1-36.5)	0.040
	Present	21	33.3 (14.9-53.1)	42.8 (21.9-62.3)	

Primary cancer status	Controlled	22	18.1 (5.7-36.3)	22.7 (8.3-41.4)	0.312
	Unconrolled	17	29.4 (10.7-51.1)	41.1 (18.6-62.6)	

Histopathology	EGFR or ALK positive lung-adenocarcinoma*	9	0	11.1 (0.6-38.8)	0.142
	Others	30	30.0 (15.0-46.6)	36.6 (20.1-53.4)	

Sum of tumor volume	<5 cc	25	16.0 (5.0-32.5)	24.0 (9.8-41.7)	0.257
	≥5 cc	14	35.7 (13.0-59.4)	42.8 (17.7-66.0)	

Max diameter of largest met	<19 mm	19	15.7 (3.9-34.9)	26.3 (9.6-46.8)	0.362
	≥20 mm	20	30.0 (12.3-50.1)	35.0 (15.7-55.2)	

No. of brain mets	1	20	25.0 (9.1-44.9)	30.0 (12.3-50.1)	0.808
	2-4	19	21.0 (6.6-41.0)	31.5 (12.9-52.2)	

Abbreviations: EGFR, Epidermal Growth Factor Receptor; ALK, Anaplastic Lymphoma Kinase.

* All patients with EGFR or ALK positive lung-adenocarcinoma received Tyrosine Kinase Inhibitor (TKI) treatment.

**Table A5:** Quality of life at baseline and after radiation therapy

	No. of patients	BL	4 mo.	8 mo.	12 mo.	BL vs. 4 mo., *p*	BL vs. 8 mo., *p*	BL vs. 12 mo., *p*
		40	34	26	18			
QLQ-C30								
Global health status/QOL*	Mean	52.1	56.6	51.9	60.6	0.424	0.979	0.226
	SD	25.6	22.5	22.0	22.3			

Functional scales*								
Physical functioning (PF)	Mean	77.5	72.2	69.2	70.0	0.186	0.142	0.287
	SD	20.4	25.0	24.5	32.1			

Role functioning (RF)	Mean	72.1	72.2	77.3	72.2	0.985	0.491	0.988
	SD	31.4	31.1	26.7	34.3			

Emotional functioning (EF)	Mean	75.0	84.8	83.0	82.9	**0.010**	0.062	0.126
	SD	17.4	14.0	15.7	18.8			

Cognitive functioning (CF)	Mean	69.6	68.6	69.2	68.5	0.841	0.948	0.862
	SD	18.8	22.0	24.8	26.7			

Social functioning (SF)	Mean	72.9	81.9	84.6	71.3	0.074	**0.033**	0.829
	SD	23.5	18.1	17.6	31.7			

Symptom scales**								
Fatigue (FA)	Mean	35.8	35.9	37.6	34.0	0.980	0.732	0.746
	SD	21.1	17.7	19.4	18.5			

Nausea and vomiting (NV)	Mean	3.8	5.4	8.7	4.6	0.531	0.294	0.734
	SD	8.0	14.0	22.1	11.2			

Pain (PA)	Mean	27.9	17.6	25.0	26.9	0.070	0.661	0.895
	SD	28.1	17.9	23.2	28.7			

Dyspnea (DY)	Mean	25.0	25.5	21.8	22.2	0.943	0.645	0.734
	SD	30.0	28.5	23.0	25.6			

Insomnia (SL)	Mean	25.8	18.6	23.1	22.2	0.199	0.674	0.640
	SD	28.7	18.7	20.6	22.9			

Appetite loss (AP)	Mean	25.0	28.3	33.3	22.2	0.632	0.273	0.751
	SD	31.8	25.2	25.5	28.0			

Constipation (CO)	Mean	24.2	32.4	15.4	9.3	0.271	0.193	0.053
	SD	28.2	35.3	23.5	22.3			

Diarrhea (DI)	Mean	10.8	16.7	16.7	13.0	0.259	0.366	0.756
	SD	21.9	22.1	30.2	28.3			

Financial difficulties (FI)	Mean	29.2	19.6	16.7	20.4	0.139	0.054	0.268
	SD	27.4	27.4	21.6	28.3			

QLQ-BN20								
Symptom scales**								
Future uncertainty (BNFU)	Mean	16.7	13.4	13.5	13.7	0.174	0.227	0.369
	SD	11.3	9.1	8.9	12.6			

Visual disorder (BNVD)	Mean	6.9	6.2	9.4	6.2	0.702	0.382	0.756
	SD	8.6	7.7	12.3	8.9			

Motor dysfunction (BNMD)	Mean	8.1	7.0	8.5	7.7	0.629	0.834	0.898
	SD	9.2	8.9	9.3	9.4			

Communication deficit (BNCD)	Mean	10.3	10.1	10.7	10.5	0.953	0.875	0.948
	SD	10.9	10.2	9.0	13.5			

Headaches (BNHA)	Mean	7.9	7.4	8.3	8.3	0.839	0.893	0.905
	SD	11.9	11.7	12.7	13.1			

Seizure (BNSE)	Mean	2.5	1.5	1.3	0.9	0.476	0.441	0.315
	SD	6.0	6.3	6.5	3.9			

Drowsiness (BNDR)	Mean	15.0	17.2	20.5	18.5	0.412	0.060	0.403
	SD	10.5	12.0	12.7	16.1			

Hair loss(BNHL)	Mean	5.8	15.2	5.1	5.6	**0.008**	0.788	0.933
	SD	11.0	17.1	9.2	12.8			

Itchy skin (BNIS)	Mean	10.0	9.3	7.1	11.1	0.842	0.439	0.808
	SD	16.4	12.4	12.6	15.1			

Weakness of legs (BNWL)	Mean	15.8	20.6	19.9	21.3	0.199	0.307	0.242
	SD	16.4	14.8	14.2	16.0			

Bladder control (BNBC)	Mean	7.9	7.4	10.3	8.3	0.804	0.347	0.885
	SD	9.2	10.2	10.6	11.8			

* Scores range from 0 to 100, with a higher score representing a higher level of functioning or health status.

** Scores range from 0 to 100, with a higher score representing a higher degree of symptoms.

**Table A6:** Number of patients with an Adverse Event by Common Terminology Criteria for Adverse Events (CTCAE) Version 4.0. Definitely or Probably Related to the Protocol Treatment

Toxicities	Grade 1	Grade 2	Grade 3	Grade 4	Grade 5
Ear and labyrinth disorders					
External ear inflammation	1	0	0	0	0
Hearing impaired	1	0	0	0	0
Middle ear inflammation	1	0	0	0	0
Gastrointestinal disorders					
Nausea	2	0	0	0	0

General disorders and administration site conditions					
Gait disturbance	0	1	0	0	0
Malaise	0	1	0	0	0

Metabolism and nutrition disorders					
Anorexia	2	1	0	0	0

Nervous system disorders					
Central nervous system necrosis	3	2	1	0	0
Cognitive disturbance	2	2	1	0	0
Headache	2	0	0	0	0
Leukoencephalopathy	5	2	1	1	0
Somnolence	0	1	0	0	0

Psychiatric disorders					
Depression	1	0	0	0	0

Skin and subcutaneous tissue disorders					
Alopecia	1	5	0	0	0
Maximum toxicity per person	**11 (27.5 %)**	**10 (25 %)**	**2 (5 %)**	**1 (2.5 %)**	**0**

## Data Availability

Research data are not available at this time.

## Appendix A

Fig. A1, Fig. A2, Fig. A3, Fig. A4, Fig. A5.

Table A1, Table A2, Table A3, Table A4, Table A5, Table A6.
